# Calcipotriol Nanosuspension-Loaded Trilayer Dissolving Microneedle Patches for the Treatment of Psoriasis: In Vitro Delivery and In Vivo Antipsoriatic Activity Studies

**DOI:** 10.1021/acs.molpharmaceut.3c01223

**Published:** 2024-05-16

**Authors:** Xianbing Dai, Andi Dian Permana, Mingshan Li, Habibie, Muhammad Nur Amir, Ke Peng, Chunyang Zhang, Haodong Dai, Alejandro J Paredes, Lalitkumar K. Vora, Ryan F. Donnelly

**Affiliations:** † School of Pharmacy, Medical Biology Centre, Queen’s University Belfast, 97 Lisburn Road, Belfast BT9 7BL, U.K.; ‡ School of Pharmacy, Jinzhou Medical University, Jinzhou, Liaoning 121001, China; § Faculty of Pharmacy, Universitas Hasanuddin, Makassar 90245, Indonesia; ∥ School of Chemistry and Chemical Engineering, Queen’s University Belfast, David Keir Building, Stranmillis Road, Belfast BT9 5AG, U.K.

**Keywords:** microneedle, nanosuspension, psoriasis, calcipotriol, nanocrystal

## Abstract

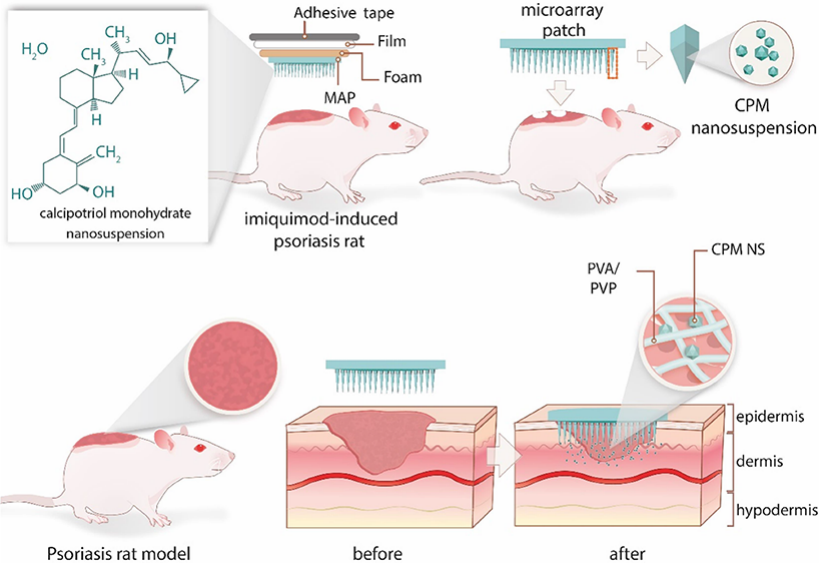

Psoriasis, affecting 2–3% of the global population, is a chronic inflammatory skin condition without a definitive cure. Current treatments focus on managing symptoms. Recognizing the need for innovative drug delivery methods to enhance patient adherence, this study explores a new approach using calcipotriol monohydrate (CPM), a primary topical treatment for psoriasis. Despite its effectiveness, CPM’s therapeutic potential is often limited by factors like the greasiness of topical applications, poor skin permeability, low skin retention, and lack of controlled delivery. To overcome these challenges, the study introduces CPM in the form of nanosuspensions (NSs), characterized by an average particle size of 211 ± 2 nm. These CPM NSs are then incorporated into a trilayer dissolving microneedle patch (MAP) made from poly(vinylpyrrolidone) and w poly(vinyl alcohol) as needle arrays and prefrom 3D printed polylactic acid backing layer. This MAP features rapidly dissolving tips and exhibits good mechanical properties and insertion capability with delivery efficiency compared to the conventional Daivonex ointment. The effectiveness of this novel MAP was tested on Sprague–Dawley rats with imiquimod-induced psoriasis, demonstrating efficacy comparable to the marketed ointment. This innovative trilayer dissolving MAP represents a promising new local delivery system for calcipotriol, potentially revolutionizing psoriasis treatment by enhancing drug delivery and patient compliance.

## 1 Introduction

Psoriasis is a chronic recurring immune-mediated disorder affecting mainly the skin, nails, and joints; has a genetic etiology, with a prevalence of approximately 2–3% worldwide; and has an increasing incidence over time.^1,2^ It is characterized by epidermal hyperplasia, desquamation, and erythema formation due to excessive proliferation and abnormal differentiation of epidermal keratinocytes.^3^ Unfortunately, the underlying pathophysiology of psoriasis is highly complex and still unclear, and psoriasis cannot be cured radically, typically requiring lifelong treatment.^4,5^ In addition, psoriasis is associated with multiple comorbidities, and patients often suffer from psychological distress, which affects their health-related quality of life.^6,7^ Approximately 80% of patients with psoriasis have moderate or mild disease and are usually treated with topical first-line therapy.^8,9^ When topical treatments are insufficient to manage the disease, systemic administration with additional potential side effects occurs.^10^ Nevertheless, even when receiving systemic therapy, patients may still need to use topical agents.^11^ If topical drug efficacy improves, fewer patients will need to begin systemic therapy, leading to a reduction in systemic exposure.^12^

Calcipotriol, a synthetic vitamin D_3_ analogue, is a commonly used topical treatment for psoriasis either in combination or as a monotherapy.^13−15^ It works by binding to the vitamin D receptor, a ligand-activated transcription factor, in target cells, such as epidermal keratinocytes and lymphocytes. In current calcipotriol treatments, drugs are delivered locally through different delivery agents, such as gels, ointments, creams, lotions, and sprays.^16,17^ The chosen vehicle has a great impact, as it influences the efficacy of the drug.^18^ Furthermore, some vehicles contain chemical enhancers and solvents, which may cause side effects, particularly when applied chronically, since many of them possess irritant properties.^19^ There are several drawbacks to current treatments, including stickiness and greasiness of the topical carriers, poor skin permeability, low retention of the drug in the skin, and lack of long-term controlled release, resulting in frequent administration and potential irritation. Patients are often dissatisfied with the delay in treatment response and the inconvenience of applying traditional topical treatments. Compared with those of healthy skin, psoriatic lesions are thicker and have greater transepidermal water loss, which limit effective transdermal delivery.^20^ Another challenge for delivery is the hydrophobic nature of calcipotriol and its low permeation through the *stratum corneum*.^21^ Therefore, developing a new drug delivery system with fewer side effects to improve patient adherence to therapies is highly desirable.

Compared to traditional formulations, nanoparticle formulations can enhance efficacy and reduce side effects by reducing the dose, dosing frequency, and dose dependence. Nanosuspensions (NSs), with a mean diameter less than 1000 nm and typical particle sizes of 200–500 nm, are produced from the therapeutic agent itself, with a minimum of surface-active agents required for stabilization.^22−24^ An increase in the surface area of NSs leads to an increase in the drug dissolution rate and saturation solubility.^25−27^ The wet media milling method, a kind of “top-down” approach, is a simple low-cost approach for formulating NSs. The pure drug was suspended in a stabilizer solution and converted from micron-sized to nanosized by mechanical energy. This process is achieved by rotating a milling chamber filled with milling media, solid drug substances, and dispersion media (stabilizer in aqueous solution) at a high rate.^28,29^ This method can be applied to small sample sizes and provides advantages in early drug development and industrial pharmaceutical production.^29−31^

To facilitate the delivery of NSs to the main target, an appropriate delivery system should be considered. As mentioned previously, conventional topical dosage forms have several drawbacks, limiting the effectiveness of calcipotriol in psoriasis treatment. To address this issue, microneedle patches (MAPs) offer an effective alternative for delivering drugs to the main target.^32,33^ MAPs are micron-sized (less than 1 mm in length), minimally invasive devices that breach the outermost layer of the skin and the *stratum corneum*, creating micron-sized pores in the skin and facilitating intradermal delivery of drugs with minimal pain and no bleeding.^34−36^ Dissolving MAPs are composed of water-soluble polymers that incorporate the drug within the MAPs themselves and dissolve once inserted into the skin.^32,37^ Dissolving MAPs can achieve controlled drug release due to the dissolution rate of the constituted polymer.^38^ In addition, dissolving MAPs often results in favorable biocompatibility and is therefore promising for long-term use.^39,40^ When poorly soluble drugs are loaded into MAP tips, drug diffusion to the drug-free polymer layer can be prevented, facilitating easy detachment of the tips from the baseplates and reducing the wearing time of the MAPs.^41,42^ Novel, trilayer MAPs were developed in this study to create a more consumer-friendly product with potentially higher user acceptability.

To develop an effective topical delivery system for psoriasis treatment, we aimed to investigate the use of CPM NSs in combination with MAPs for rapid drug deposition as well as localized and sustained intradermal delivery. To the best of our knowledge, this is the first study in which a CPM NS was developed using the wet bead milling method. Additionally, this is the first instance of developing CPM-loaded dissolving MAPs. The only previous work in the area involved the combination of pretreatment with drug-free MAPs made from poly(lactic acid) and hyaluronic acid, followed by the application of CPM ointment.^43,44^

## 2 Materials and Methods

### 2.1 Materials

Calcipotriol monohydrate was kindly gifted by LEO Pharma A/S, Ballerup, Denmark. Poly(vinyl alcohol) (*M*_w_ = 9–10 kDa, 80% hydrolyzed) (PVA 10 K) was purchased from Sigma–Aldrich (Dorset, UK). Poly(vinylpyrrolidone) (PVP) (*M*_w_ = 58 kDa) was obtained from Ashland (Kidderminster, UK). Imiquimod cream (5%, Aldara) was obtained from MEDA Pharmaceuticals GmbH & Co. KG, Bad Homburg, Germany. Dovonex ointment was obtained from LEO Pharma A/S, Ballerup, Denmark. Purified water was obtained using a water purification system (Elga PURELAB DV 25, Veolian Water Systems, Ireland). All the other reagents used were of analytical grade.

### 2.2 Preparation and Lyophilization of NSs

**Figure 1:**
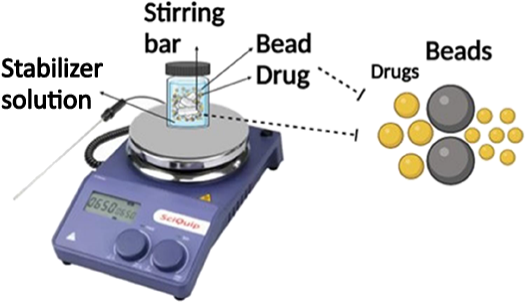
Schematic illustration of the process of fabricating NSs.

CPM NSs were prepared via a “top-down” small-scale wet bead milling method.^45^ Briefly, the milling chamber was charged with milling media (four 12 × 6 mm cylindrical magnetic stirring bars and 2 mL of 0.1 mm diameter ceramic beads (Chemco Advance Material Co., Ltd., Suzhou, China), CPM, and 7 mL of stabilizer (2% PVA 10 K) solution and agitated by a magnetic stirrer plate at a speed of 1500 rpm, as shown in Figure 1. After that, the NSs were filtered, prefrozen in a −80 °C freezer, and lyophilized by a VirTis AdVantage Plus Freeze Drier (SP Scientific Industries, Inc., New York, USA) for 25 h.

### 2.3 Characterization of NSs

#### 2.3.1 Morphology of the Pristine Drug and NSs

The morphologies of the bulk CPM and lyophilized CPM NSs were observed using a Tabletop TM 3030 scanning electron microscope (Hitachi, Tokyo, Japan).

#### 2.3.2 Particle Size Determination for the Pristine Drug and NSs

Before preparing NSs by wet bead milling, the particle size of the pristine drug was determined by laser diffraction using a Mastersizer 3000 equipped with a Hydro EV Flexible volume wet dispersion cell (Malvern Panalytical Ltd., Worcestershire, UK) six times.^26^ The particle size and polydispersity index (PDI) of freshly prepared NSs and lyophilized NSs were determined by dynamic light scattering (DLS) using a NanoBrook Omni analyzer (Brookhaven, New York, USA). Measurements were conducted at an equilibration time of 3 min at a temperature of 25 °C. All the measurements were performed in triplicate, and the obtained results were compared to the initial characteristics.

#### 2.3.3 NS Stability

The short-term physical temperature stabilities of the NSs were assessed at −80 °C and room temperature in the dark. The effect of light was evaluated at room temperature. After quick manual redispersion, 20 μL of NSs was dispersed in 3 mL of water, and the particle size and PDI were measured as described above. The stability of the lyophilized NSs was also determined.

#### 2.3.4 Attenuated Total Reflectance Fourier Transform Infrared Spectroscopy (ATR-FTIR)

Infrared spectroscopic patterns of CPM, PVA, and lyophilized CPM NSs and their physical mixtures (with the same proportions as lyophilized NSs, CPM, and PVA at 5/7, w/w) were obtained by an FTIR Accutrac FT/IR-4100 Series (Jasco, Maryland, USA) instrument equipped with a MIRacle diamond ATR accessory (Pike Technologies, Madison, Wisconsin). The absorption spectra were recorded from 4000 to 600 cm^–1^, and the resolution used in the scans was 4 cm^–1^ at 32 scans per spectrum.

#### 2.3.5 Differential Scanning Calorimetry (DSC)

The thermal analyses of CPM, lyophilized CPM NSs, PVA, and physical mixtures were subjected to DSC analysis. Samples (3–4 mg) were accurately weighed, sealed in standard aluminum pans, and scanned on a Q100 differential scanning calorimeter (TA Instruments, Delaware, USA) with an empty standard aluminum pan as a reference. DSC analysis was conducted at 10 °C/min from 30 to 300 °C under 50.0 mL/min nitrogen flow.

#### 2.3.6 Powder X-ray Diffraction (XRD)

The crystallinity of the lyophilized CPM NSs and the raw formulation materials was analyzed on a Bruker D8 Advance diffractometer (Bruker, Inc., Karlsruhe, Germany) in reflection mode with Cu–Kα radiation (λ = 1.5418 Å) at a voltage of 40 kV and a current of 25 mA. All the samples were scanned using continuous scan mode with a step width of 0.01° (2θ) and 1 s per step, varying from 5 to 50° (2θ).

#### 2.3.7 In Vitro Drug Release Evaluation

The in vitro drug release profile of lyophilized CPM NSs was determined via diffusion studies using a dialysis method.^4,46^ Due to the intrinsic hydrophobicity of CPM, 2-propanol was added to the PBS solution to achieve sink conditions. Therefore, the release medium was composed of PBS (pH = 7.4) and 2-propanol at a ratio of 70:30 v/v. The dialysis membrane was cut to size and soaked in release medium at 37 ± 0.5 °C for 0.5 h prior to equilibration. In triplicate, either lyophilized CPM NSs (equivalent to 5 mg of CPM) or the CPM bulk drug were dispersed in 3 mL of the release medium and loaded into a Spectra-Por dialysis bag with a molecular weight cutoff (MWCO) of 12,000–14,000 Da. The dialysis bag was then placed in a hermetically sealed 500 mL bottle filled with the release medium. The bottles were placed at 37 °C in an orbital shaker at 80 rpm for 21 days. Aliquots of 1 mL of sample were collected and replaced with 1 mL of prewarmed fresh release medium at the desired time. To calculate the percentage of each drug released over time, the concentration of each drug in the withdrawn samples was then analyzed via HPLC.

### 2.4 Characterization of Dissolving MAPs

#### 2.4.1 Preparation of MAPs

**Figure 2:**
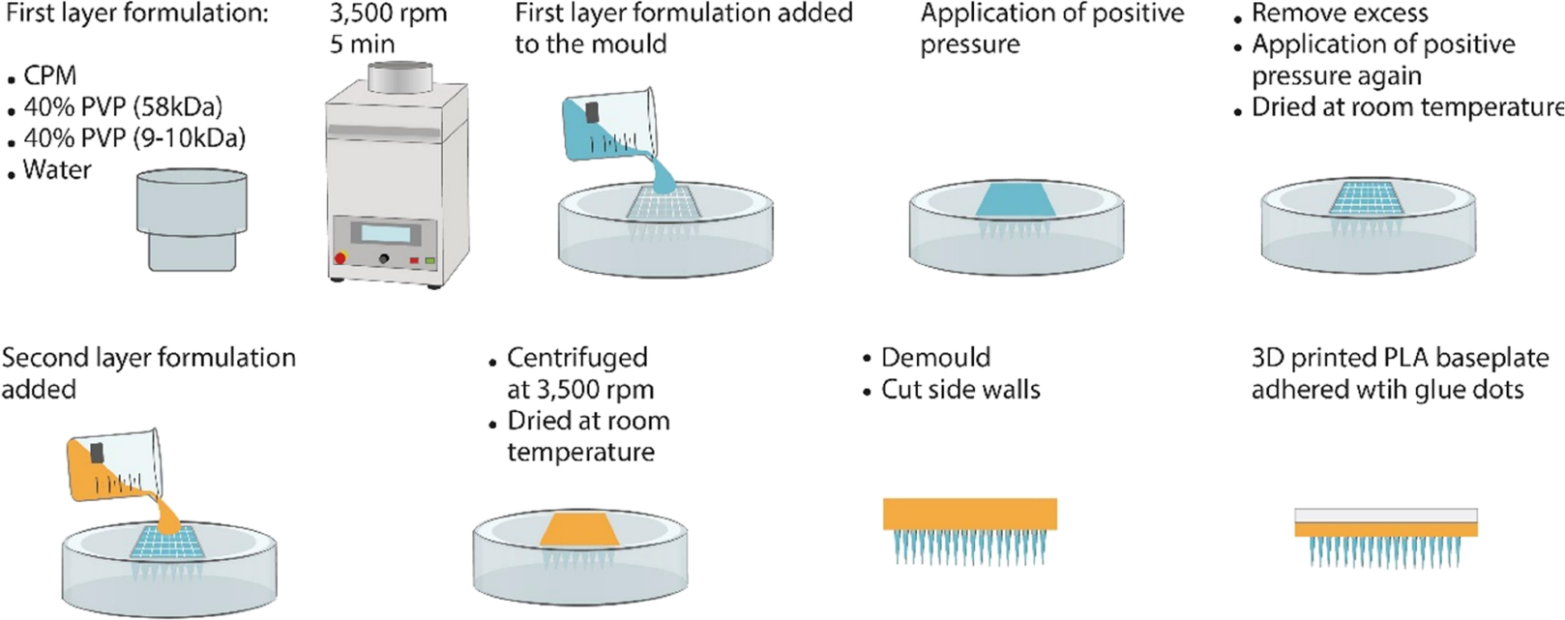
Schematic illustration of the fabrication process of dissolving MAPs.

Trilayer CPM MAPs were fabricated by a micromolding technique. Initially, 20 mg of CPM NSs was mixed with 1570 mg of an aqueous mixture of 40% w/w poly(vinylpyrrolidone) (PVP) (*M*_w_ = 58 kDa) and 40% w/w poly(vinyl alcohol) (PVA) (*M*_w_ = 9–10 kDa) at a ratio of 1:1 in a SpeedMixer (Hauschild, Hamm, Germany) at 3500 rpm for 3 min.^47^ The formulation was poured into silicone MAP molds (600 pyramidal needles on a 0.76 cm^2^ area with a 750 μm needle height), placed in a positive pressure chamber (5 bar was applied for 3 min), and subsequently placed in the pressure chamber again for another 3 min at 5 bar. After drying at room temperature overnight, 0.25 g of PVP–PVA drug-free aqueous solution (40 wt % PVP, 40 wt % PVA) was added, and the mixture was centrifuged at 3500 rpm for 10 min. After drying, the PLA 3D-printed solid baseplates were adhered to the back of the MN patches with glue dots.^48^ The process of preparing the CPM powder MAPs and the formulation composition used were identical to those described above for the CPM NS MAPs (Figure 2).

#### 2.4.2 Morphology of the CPM MAP

The morphologies of the CPMs were observed using a Tabletop TM 3030 scanning electron microscope (Hitachi, Tokyo, Japan) and a Leica EZ4 D digital microscope (Leica Microsystems, Milton Keynes, UK).

#### 2.4.3 Mechanical Performance and Insertion Behavior of the MAP

The mechanical and insertion properties were determined by attaching a MAP to a cylindrical probe of a TA. An XT2 texture analyzer (Stable Micro Systems., Ltd., Haslemere, UK) in compression mode was used as previously described.^35,45^ The probe was moved vertically downward at a speed of 0.5 mm/s to compress the MAP with a force of 32 N for 30 s. A flat aluminum block was used against the MAP to test its mechanical properties. Before and after compression, the length of each individual needle in the MAPs was measured. Eight layers of Parafilm M (approximately 1 mm thick) were used as the skin simulant model to evaluate the insertion behavior. The holes created in each layer were counted under observation with a Leica EZ4 W stereomicroscope (Leica Microsystems, Milton Keynes, UK). To gain more insights into the insertion behavior of MAPs into the skin, an EX1301 optical coherence tomography (OCT) microscope (Michelson Diagnostics Ltd., Kent, UK) was used to visualize the insertion behavior of MAPs in full-thickness neonatal porcine skin, a well-established model of human skin. Before use, the porcine skin was equilibrated in PBS (pH = 7.4) for 30 min at 37 °C and then shaved.

#### 2.4.4 Dissolution Test of MAPs in Excised Porcine Skin

The dissolution of MAPs was determined by taking images at different time points using a Leica EZ4 D digital microscope (Leica Microsystems, Milton Keynes, UK). The MAPs were inserted into excised porcine skin and placed on a tissue paper soaked in PBS. The samples were subsequently incubated in an oven set at 37 °C to mimic and maintain the body temperature.^49^

#### 2.4.5 Determination of CPM in Whole MAPs, MAP Tips, and Baseplates

The tips and baseplate of the MAPs were carefully scraped with a scalpel. The tips and baseplate of each MAP and each complete MAP were fully dissolved in 5 mL of deionized water using magnetic stirring for 15 min. Subsequently, this solution was mixed with 5 mL of menthol, agitated for an additional 15 min, filtered, and subjected to HPLC analysis.^50^

#### 2.4.6 Drug Content Stability Tests of MAPs

The drug content of the entire set of MAPs was determined to assess the stability of the NS MAPs and powder MAPs over a period of 6 months.

#### 2.4.7 Ex Vivo Skin Permeation and Retention Study

To evaluate drug permeation and deposition in the skin, a Franz cell setup was used. Ex vivo skin permeation and deposition studies were also conducted on full-thickness neonatal porcine skin. The skin was glued on the donor part of static vertical glass Franz diffusion cells. A CPM MAP was applied to the skin manually for 30 s, after which a 12 g weight was placed on top of the MAP to hold it in place. For comparison, 100 mg of the marketed CPM ointment Dovonex (50 μg/g CPM) was used. The release media used for the receptor were PBS (pH = 7.4) and 2-propanol at a ratio of 70:30 v/v. The temperature of the receiver compartment was maintained at 37 ± 1 °C, and the mixture was continually stirred at a speed of 600 rpm. After equilibration, the donor compartment was placed on the receiver compartment. The donor compartment and applied MAPs were removed at predefined time intervals. The baseplates were then carefully removed, and the skin surface was cleaned three times with PBS (pH = 7.4) solution and gently dried. After that, the skin was observed with a Leica EZ4 W stereomicroscope at the application location and then harvested by an 8.0 mm diameter biopsy punch (Stiefel, Middlesex, UK). Next, the skin samples were cut into tiny pieces with scissors and homogenized with stainless steel beads by TissueLyserLT (QIAGEN, U.K.) to extract the drug as previously described.^34^ The drugs in the skin and in the receiver were quantified by HPLC.

### 2.5 In Vivo Studies

To further evaluate the antipsoriatic efficacy of CPM MAPs, MAPs were applied to imiquimod (IMQ)-treated Sprague–Dawley rats with psoriasis-like skin lesions, a commonly used suitable animal model for short-term psoriasis animal studies. For comparison, rats suffering from psoriasis received blank MAPs without CPM, marketed CPM ointment, or healthy controls.

#### 2.5.1 Groups and Treatments

The in vivo antipsoriatic activity experiments were conducted in accordance with the Guiding Principles for the Care and Use of Laboratory Animals, and the experimental protocol used in this study was approved by the Committee for Animal Experiments at Hasanuddin University under Project License No. 201022105032.

Healthy female Sprague–Dawley rats (11–12 weeks of age) weighing 233.03 ± 40.24 g were obtained from the Faculty of Pharmacy, Hasanuddin University. The rats were kept in a specific pathogen-free animal facility with a maintained temperature of 19–25 °C, humidity of 30–70%, and a 12 h day/night cycle and were given access to food and water *ad libitum*.

**Figure 3:**
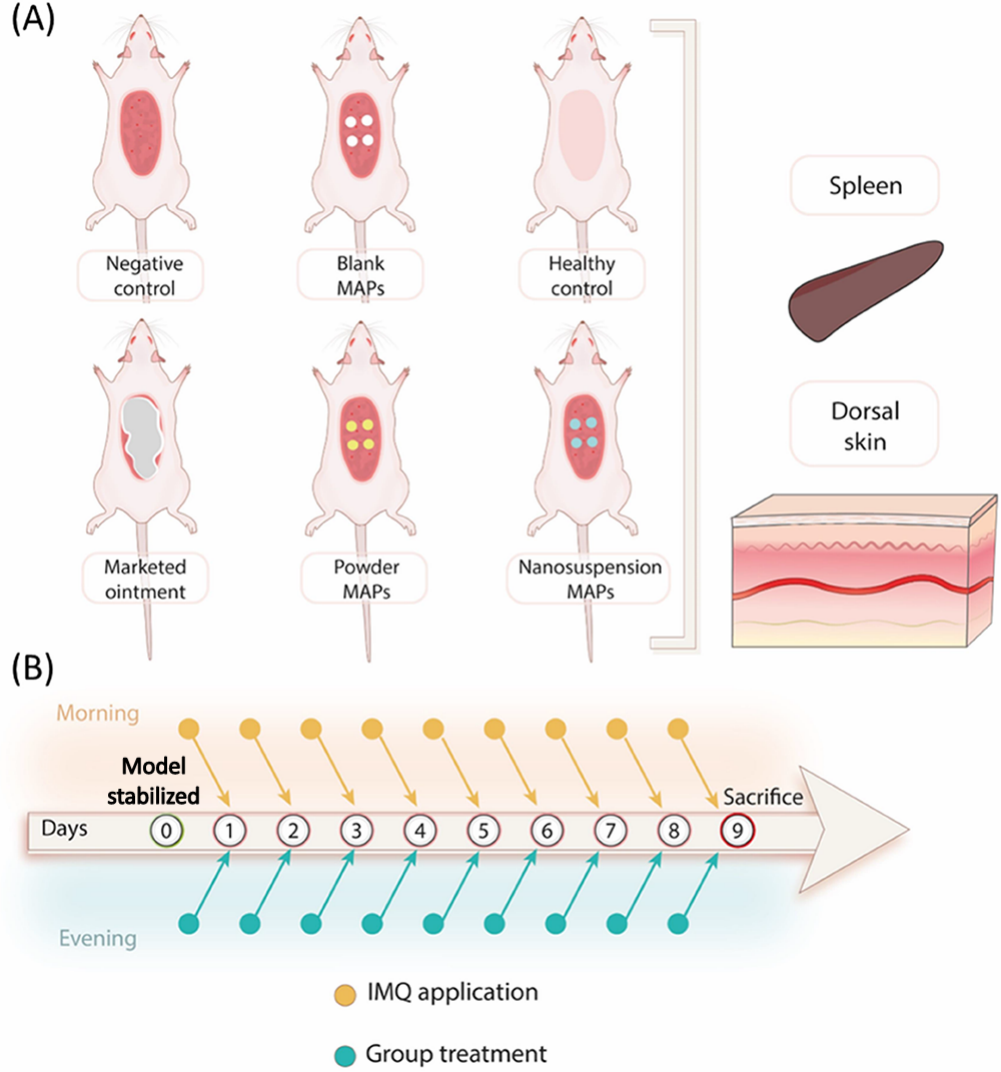
(A) Diagrammatic illustration of the six treatment cohorts investigated in the in vivo experiments. The skin and spleen were excised after the animals were sacrificed for the investigation of antipsoriatic activities. Created with BioRender.com. (B) Schematic illustration of the treatment schedule for psoriasis. After the establishment of the model, IMQ was applied every morning, and the treatments were applied every evening. Created with ProcessOn.com.

As illustrated in Figure 3, the animals were randomly assigned into six groups, with each group comprising five animals. Group I served as the negative control, where the disease was induced using IMQ. Group II consisted of animals treated with blank MAPs, while Group III used the marketed ointment Dovonex as a positive control. Group IV received treatment with Powder MAPs, and Group V was treated with NS MAPs. Group VI, the sham group, consisted of healthy animals. For all groups, the hair on the backs of the animals was carefully removed to facilitate treatment application and observation. For all groups, the hair on the backs of the animals was first shaved using an electric shaving razor, and then, Veet sensitive skin hair removal creams were applied for 6 min, followed by removal with absorbent paper and water a day prior to initiation of the study. The rats were left to recover overnight, and no adverse effects were observed during the monitoring period. Psoriasis was induced by the once daily application of 5% IMQ cream to an area of 10 cm^2^ on the back skin of the rats. Cream (50 mg) was applied in the morning to the shaved back of each rat in each group except for the sham group, which was the healthy control rat group (Group VI). During the treatment period, rats were administered imiquimod (IMQ) in the morning and received treatments in the evening (Group I–Group V). The rats in the negative control group (Group I) received IMQ only in the morning and were not given any other treatments; these rats comprised the disease-induced control group. For therapies, the marketed CPM ointment Dovonex containing 50 μg/g CPM was applied once daily as a positive control (Group III) for 8 consecutive days in the evening. Similarly, in the MAP application groups (Group II, Group IV, and Group V), four patches of MAPs were applied once daily in the evening. After 9 days, the animals were sacrificed, and the skin and spleen were subsequently excised from each animal.

#### 2.5.2 Body Weight Changes

The body weights of each rat in all groups were recorded every day during the experiment. The percentage change in body weight was calculated as the ratio of the measured weight to the initial weight.

#### 2.5.3 Morphological Changes in Skin

##### 2.5.3.1 Psoriasis Area and Severity Index (PASI) Score

The severity of psoriasis in the rat skin was scored based on the PASI score.^51^ Three parameters, namely, erythema (redness, red, or dark red inflammatory spots that fade under pressure), induration (thickness, the tendency of skin lesions to spread around, with blurred boundaries and a sense of substance), and desquamation (scaling, peeling off epidermal cells in pieces), were scored independently from 0 to 4 as follows: 0, none; 1, slight; 2, moderate; 3, marked; and 4, very marked. The cumulative score (erythema plus induration plus desquamation) indicated the total severity of inflammation (scale 0–12). The PASI was calculated every day during the whole study period.

##### 2.5.3.2 Skin Thickness

Changes in skin thickness are a visual indicator of hyperproliferation and abnormal differentiation of epidermal keratinocytes, as well as inflammation, which are the characteristic features of psoriasis.^52,53^ The skin thickness was measured by a digital caliper after the animals were sacrificed.

##### 2.5.3.3 Skin Moisture

In parakeratotic psoriatic plaques, the protective function of the outermost layer of the skin (known as the *stratum corneum*) is compromised, resulting in greater water loss in these areas than in healthy skin.^54^ Consequently, psoriatic skin lesions have less skin moisture than normal skin. Skin moisture was measured by a skin moisture analyzer (Skin Analyzer VCare Mode SK-8, Vcare, India) after the animals were sacrificed.

##### 2.5.3.4 Histopathological Analyses

At the end of the experiment, the back skin from all groups was isolated and examined macroscopically. The tissues were fixed in 10% neutral buffered formalin (Sigma-Aldrich, Singapore), embedded in paraffin (Sigma-Aldrich, Singapore), sliced into 250 μm thick sections, stained with hematoxylin and eosin (H&E) (Sigma-Aldrich, Singapore), and observed under a light microscope (Olympus).

#### 2.5.4 Spleen Morphological Changes

On day 9, the rats were sacrificed, and their spleens were excised to study the morphological changes. The spleen length and weight were measured and recorded. The spleen index was calculated as the percentage of the spleen weight relative to the total body weight.

### 2.6 Reverse-Phase High-Performance Liquid Chromatography (RP-HPLC) Analysis

RP-HPLC-UV was used to quantify the amount of CPM. The analysis was performed using a Phenomenex SphereClone 5 μm (150 × 4.60 mm) column (Phenomenex, Cheshire, UK) and an Agilent Technologies 1260 Infinity II series HPLC system with UV detection (Agilent Technologies UK Ltd., Stockport, UK). The maximum absorption (λ_max_) was fixed at 265 nm, and the injection volume was 50 μL. The mobile phase was composed of 0.1% (v/v) triethylamine in water (adjusted to pH = 6 with 85% phosphoric acid) and methanol (20:80, v/v) at a flow rate of 1 mL/min at 25 °C. This method was validated based on the 2005 International Committee on Harmonization (ICH) guidelines.^55^ The linearity of the method was explored between 0.05 and 20 μg/mL (*R*^2^ = 0.9999), with a limit of detection (LoD) of 0.15 μg/mL and a limit of quantification (LoQ) of 0.47 μg/mL.

### 2.7 Statistical Analysis

In this study, all the experiments were performed on at least three replicate samples. All the data are expressed as mean ± standard deviation (SD). The data were analyzed using GraphPad Prism version 9 (GraphPad Software, San Diego, California, USA). Differences between two groups were assessed using Student’s *t* test, and multiple comparisons among different groups were made using one-way analysis of variance, followed by Tukey’s multiple tests. In all the cases, *p* < 0.05 was considered to indicate a significant difference. Differences were regarded as significant if *p* < 0.05* and *p* < 0.001***.

## 3 Results and Discussion

### 3.1 Development and Short-Term Physical Stability of CPM NSs

**Figure 4:**
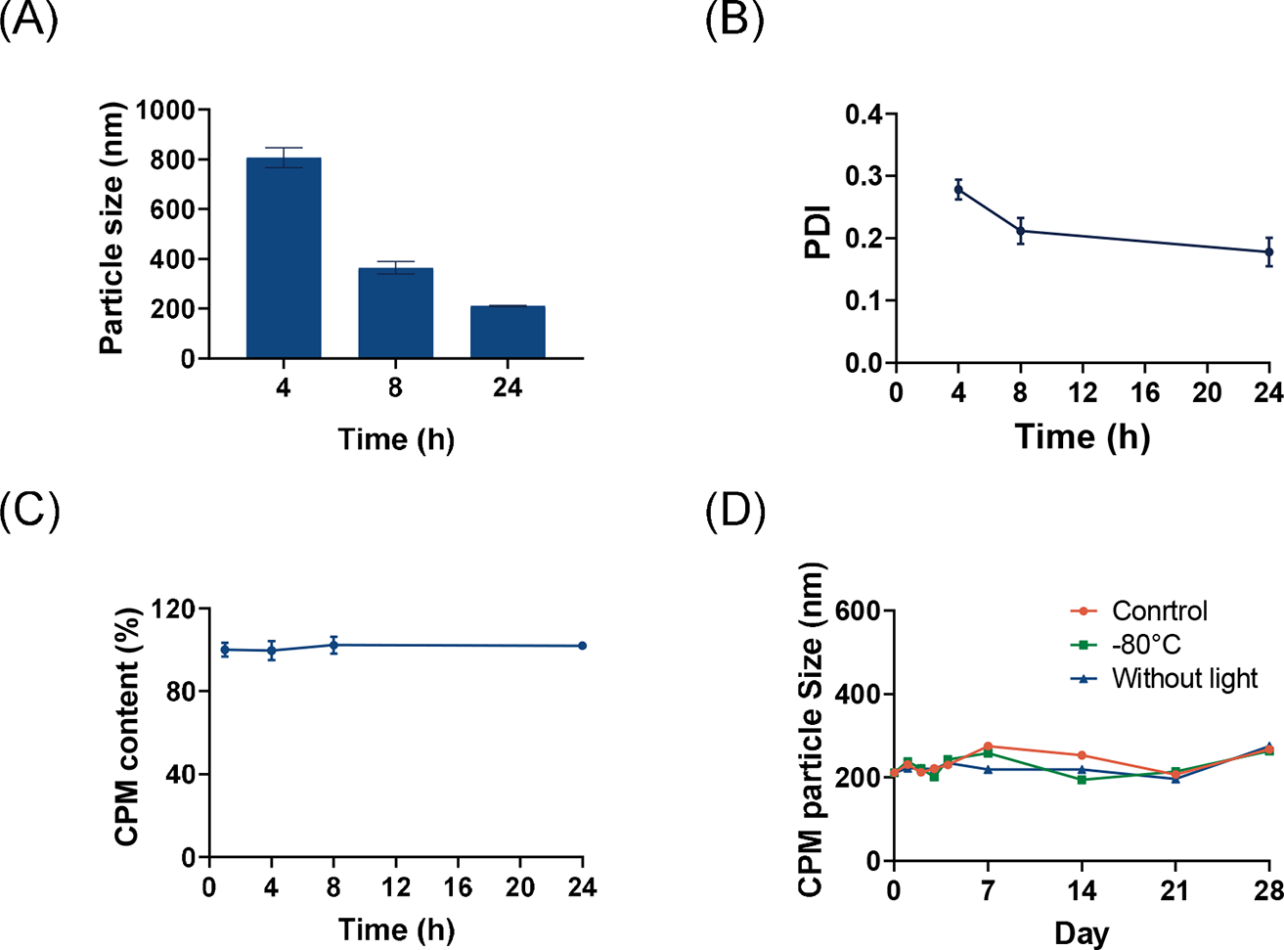
(A) Particle size of the CPM NSs at different time points (mean + SD, *n* = 3), (B) PDI of the CPM NSs at different time points (mean ± SD, *n* = 3), (C) changes in the CPM content during the NS milling progress (mean ± SD, *n* = 3), and (D) changes in the particle size stability of the CPM NSs under different storage conditions over a period of 28 days (mean ± SD, *n* = 3).

The selection of the stabilizer type and concentration is a crucial step in successfully forming drug nanoparticles, followed by milling time, medium size and amount, speed, and amount of drug. The choice of stabilizer should be based not only on its stabilizing effect and the properties of the drug NSs but also on the properties of the final product. CPM NSs were incorporated into dissolving polymeric MAPs in subsequent studies, and the NSs should be appropriate for MAP manufacturing. Polymers are among the many stabilizers whose hydrophilic chain length, structure, and molecular weight can affect their efficiency in stabilizing NSs.^56^ Accordingly, PVA 10 K and PVP (58 kDa) were selected as the stabilizers because they are also used in formulating MAPs that function as matrices, ultimately reducing the total amount of excipient needed.^57^ Furthermore, the nanoparticles stabilized by PVA 10K are compatible with MAPs.^49^ Initially, a 1% (w/w) PVA 10 K solution was used as a stabilizer for the preparation of CPM NSs. However, the particle size distribution was quite broad, and often, the desired nanosize was not achieved (data not shown). Subsequently, a 2% (w/w) PVA 10 K solution was utilized as a stabilizer, and the milling process was carried out using 100 mg of CPM (Figure 4A,B). The drug content remained nearly the same during the milling process (Figure 4C). The whole process of milling did not cause CPM degradation. Therefore, the CPM NSs were formulated in a cost-effective and simplified manner. After milling for 24 h, the particle size of the finalized CPM NSs was 211 ± 2 nm, and the PDI was 0.18 ± 0.02.

NSs can be thermodynamically unstable due to their small particle size, which can lead to particle size growth caused by Ostwald ripening.^58,59^ This process can cause a broadening of the particle size distribution over time and a decrease in the stability and efficacy of the NSs. Therefore, it was crucial to assess the impact of environmental stresses on NS particle properties to identify appropriate storage conditions and the maximum duration allowed before lyophilization. During a storage period of 28 days, the particle size of the formulated CPM NSs remained below 280 nm with/without light or at −80 °C. This demonstrated the stability of our formulation at room temperature in the dark (Figure 4D)

### 3.2 Lyophilization and Characterization of CPM NSs

To concentrate the nanoparticle dispersion, maintain particle size during storage, and prolong self-life, the CPM NSs were lyophilized after quick prefreezing. The freshly prepared CPM NSs were milky white in appearance. The freeze-dried NSs were easily resuspended in water with a short reconstitution time and maintained their adequate appearance when freshly prepared.

#### 3.2.1 Particle Size Determination and SEM Images of the Crude Drug and NSs

**Figure 5:**
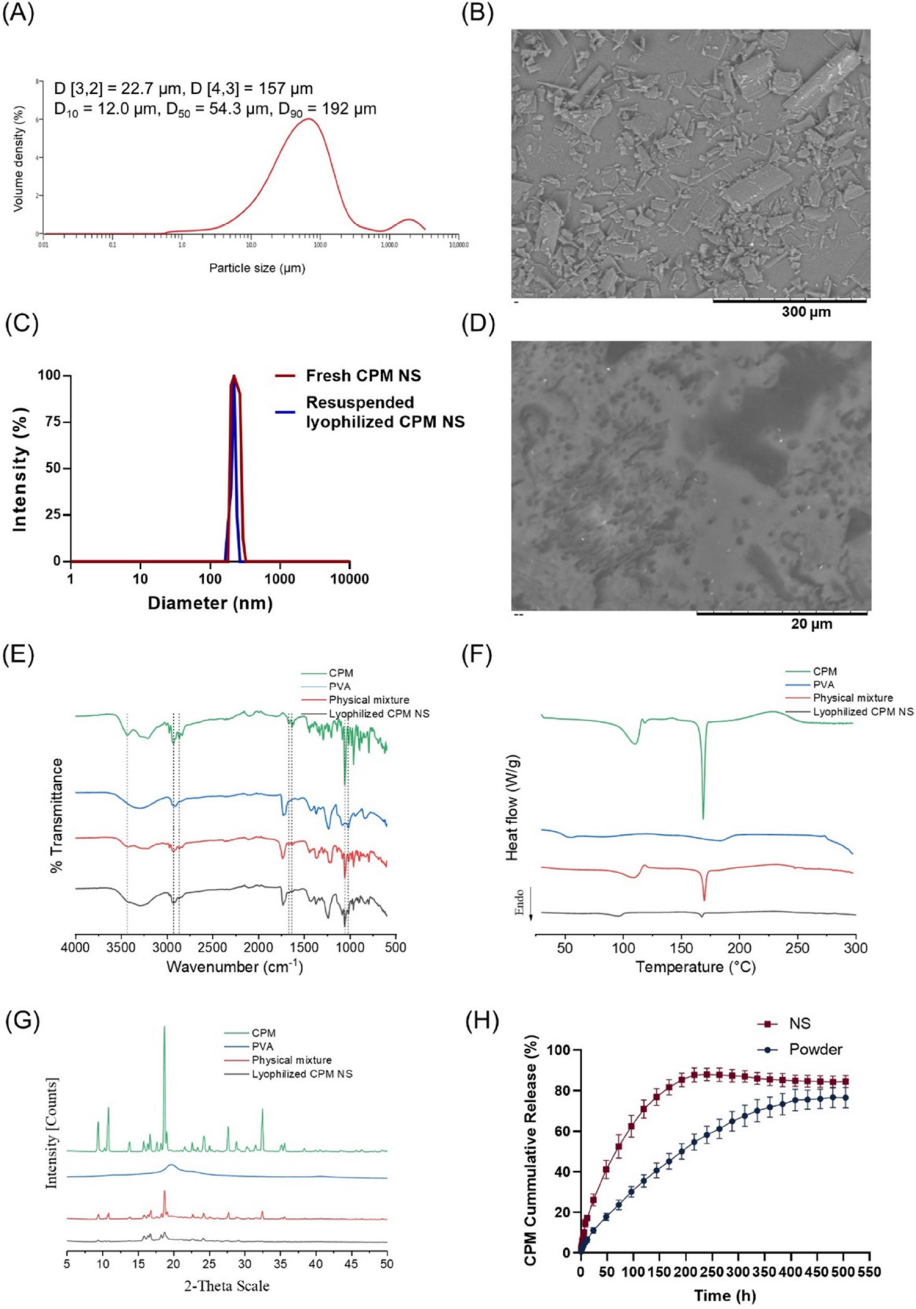
(A) Distribution profile of crude CPM (mean, *n* = 6), (B) SEM images of crude CPM powder, (C) DLS reports of the CPM NSs, and (D) SEM images of lyophilized CPM NSs. (E) FTIR, (F) DSC, and (G) XRD data of bulk CPM powder, 9–10 kDa poly(vinyl alcohol), a physical mixture of CPM and PVA (at 5/7, w/w) and lyophilized CPM NSs. (H) In vitro cumulative release profiles of lyophilized CPM NSs and bulk CPM powder (mean ± SD, *n* = 3).

According to the reports presented in Figure 5C for CPM NSs, the mean particle size and PDI remained nearly the same at a narrow size distribution before and after lyophilization without any need for any lyoprotectant (*p* > 0.05). Before lyophilization, the mean particle size and PDI were 211 ± 2 nm and 0.15 ± 0.03, respectively, while after lyophilization, these values were 195 ± 1 nm and 0.14 ± 0.03, respectively. A comparison of the results shown in Figure 5A,C revealed that the particle size of the crude drug powder was greater than that of the other powders, and the distribution profile of the crude drug powder was wider (*D* [4,3] = 157 μm, *D* [3,2] = 22.7, and *D*_90_ = 54.3). The crude drug powder and lyophilized NSs were viewed via a SEM system (Figure 5B,D). The lyophilized NSs contained individual white dots, which were assumed to be drug nanoparticles. In comparison to the wide distribution and needle-like shape of the crude drug, the lyophilized NSs exhibited uniform dot-like nanoparticles. The particle sizes of both the crude drug and lyophilized NSs observed through SEM were consistent with the particle size measurements obtained from Mastersizer 3000 and DLS systems.

#### 3.2.2 ATR-FTIR, DSC, and XRD of Lyophilized CPM NSs

During the preparation of NSs, amorphous and polymorphic changes may occur, although water can stabilize the crystalline state.^56^ ATR-FTIR, DSC, and XRD were applied to determine the compatibility of CPM with the stabilizer (PVA) and the physicochemical state of the CPM NSs.

The ATR-FTIR spectra of the physical mixture and lyophilized CPM NSs exhibited no changes in the molecular structure of the CPM (Figure 5E). The absorption peak at 3433 cm^–1^ was attributed to the O–H stretching vibration, while the peaks at 2927 and 2867 cm^–1^ were attributed to C–H stretching. C=C stretching vibrations were observed at 1671 and 1637 cm^–1^. The peaks at 1058 and 1017 cm^–1^ were attributed to the C–O stretch. These findings are in agreement with the literature.^60^

In this study, DSC thermograms of bulk CPM and PVA 9–10 kDa as well as their physical mixture and lyophilized CPM NSs were obtained, as shown in Figure 5F. Since CPM is a monohydrate, its water content peak was 110.5 °C, followed by another single, strong, sharp endothermic peak at 168.83 °C, which is related to the melting point of CPM. The thermograms indicated a typical crystalline form of calcipotriol monohydrate, in agreement with previously reported literature.^61^ Lyophilized CPM NSs and the physical mixture exhibited sharp endothermic peaks at 167.68 and 169.8 °C, respectively. The water peaks were at 96.51 and 109.74 °C. Both these intense peaks corresponded to the melting point of the CPM drug crystals, suggesting the presence of a crystalline form of CPM, and no interactions were formed between the ingredients. There were no new peaks present, indicating that no new solid phases were formed. The reason for the peak advance of the lyophilized NSs was the smaller particle size. The decreases in peak height and enthalpy may be attributed to the lower drug content in the NSs and the physical mixture that could be placed in the aluminum pans compared to that in the pure drug. Another contributing factor is the decrease in particle size and crystallinity. The thermograms indicated that CPM remained in crystalline form in its respective lyophilized NSs without any chemical interactions, and no solid phase formed, consistent with the ATR-FTIR spectral study findings.

The powder X-ray diffraction (XRD) patterns of bulk CPM, PVA, the physical mixture, and lyophilized CPM NSs are displayed in Figure 5G. Bulk CPM exhibited a high degree of crystallinity with intense, narrow, sharp, and well-defined peaks at 9.39, 16.66, 18.68 and 24.22°, respectively. The positions of the XRD patterns of the lyophilized NSs (9.41, 16.71, 18.69, and 24.16°) were quite identical to those of the physical mixture as well as bulk CPM, with decreased intensity peaks, and no new peaks were found, indicating that CPM remained in crystalline form with reduced crystallinity. This was consistent with the DSC study results.

#### 3.2.3 Drug Content and Stability of Lyophilized NSs

The drug content of the lyophilized CPM NSs was 0.36 ± 0.02 w/w, which equates to a percentage recovery of 87.02 ± 4.32% w/w (Table 1). Drug loss could occur because some drugs may be attached to the milling beads during the filtering process.^60^ After 3 months, there was no significant difference in the drug content of the lyophilized CPM NSs (*p* = 0.8593), indicating that they can be stored for up to 3 months.

**Table 1:** Drug Content of Lyophilized CPM NSs Over 3 Months (mean + SD, *n* = 9)

CPM NS drug content (w/w)	0 month	3 months
amount	0.36 ± 0.02	0.35 ± 0.03
percentage recovery	87.02 ± 0.04%	85.82 ± 0.07%

#### 3.2.4 In Vitro Drug Release Study

The in vitro release profiles of bulk CPM and lyophilized CPM NSs obtained by dialysis are illustrated in Figure 5H. In comparison to the bulk drug form, lyophilized CPM NSs improved the overall cumulative dissolution release. The profile of the CPM bulk drug exhibited a slower dissolution rate—only 11.07 ± 1.4% after 24 h—whereas 26.12 ± 2.93% of the CPM was released from the lyophilized NSs—more than 2 times greater than that of the bulk drug at 24 h. On day 10, the lyophilized CPM NSs achieved 87.95 ± 3.10% cumulative release, while on day 21, the final cumulative release was 76.67 ± 5.03%. The improved dissolution rate was caused by the reduction in particle size and subsequently an increase in surface area as well as thickness of the diffusion layer of the drug crystal and an increase in saturation solubility, according to the Noyes–Whitney equation.^62^

### 3.3 Development and Fabrication of Trilayer MAPs

The bottom of the MAP tips cannot be fully inserted into the skin. To efficiently deliver drugs and potentially reduce drug waste in MAPs, the drug was designed to be loaded only into the tip layer.^63,64^ To avoid drug diffusion to the baseplate and reduce wearing time, only a small amount of drug-free polymer was used for the second layer to connect with the MAP tips and 3D-printed supported third layer. This trilayer design could allow short wearing times to completely detach the dissolving tips from the baseplate.^48,65^ Facilitated tip detachment can potentially reduce wearing time and allow the delivery of the drug intradermally in a short duration of MAP application to the patient, consequently improving patient compliance.

MAP tips were prepared using aqueous blends of PVA and PVP as matrices due to their established safety, biocompatibility, mechanical strength, and affordability.^47,64^ Low-molecular-weight polymers (<60 kDa) can be cleared by the kidneys and thus do not accumulate in the body.^66^ The combination of PVA and PVP provided good mechanical characteristics, plasticity, and dissolvability to the MAPs.^67^ MAPs fabricated solely with PVA exhibit easy bendability, whereas MAPs fabricated solely with PVP display brittleness. The combination of PVP and PVA has the potential to achieve excellent mechanical properties in MN patches.^49,68^ This difference is attributed to the strong hydrogen-bonding interactions between the −OH groups of PVA and the −C=O groups in the pyrrolidone rings of PVP. Thus, PVP–PVA blending occurs, resulting in good mechanical characteristics.^57,69^

#### 3.3.1 Morphology of MAPs

**Figure 6:**
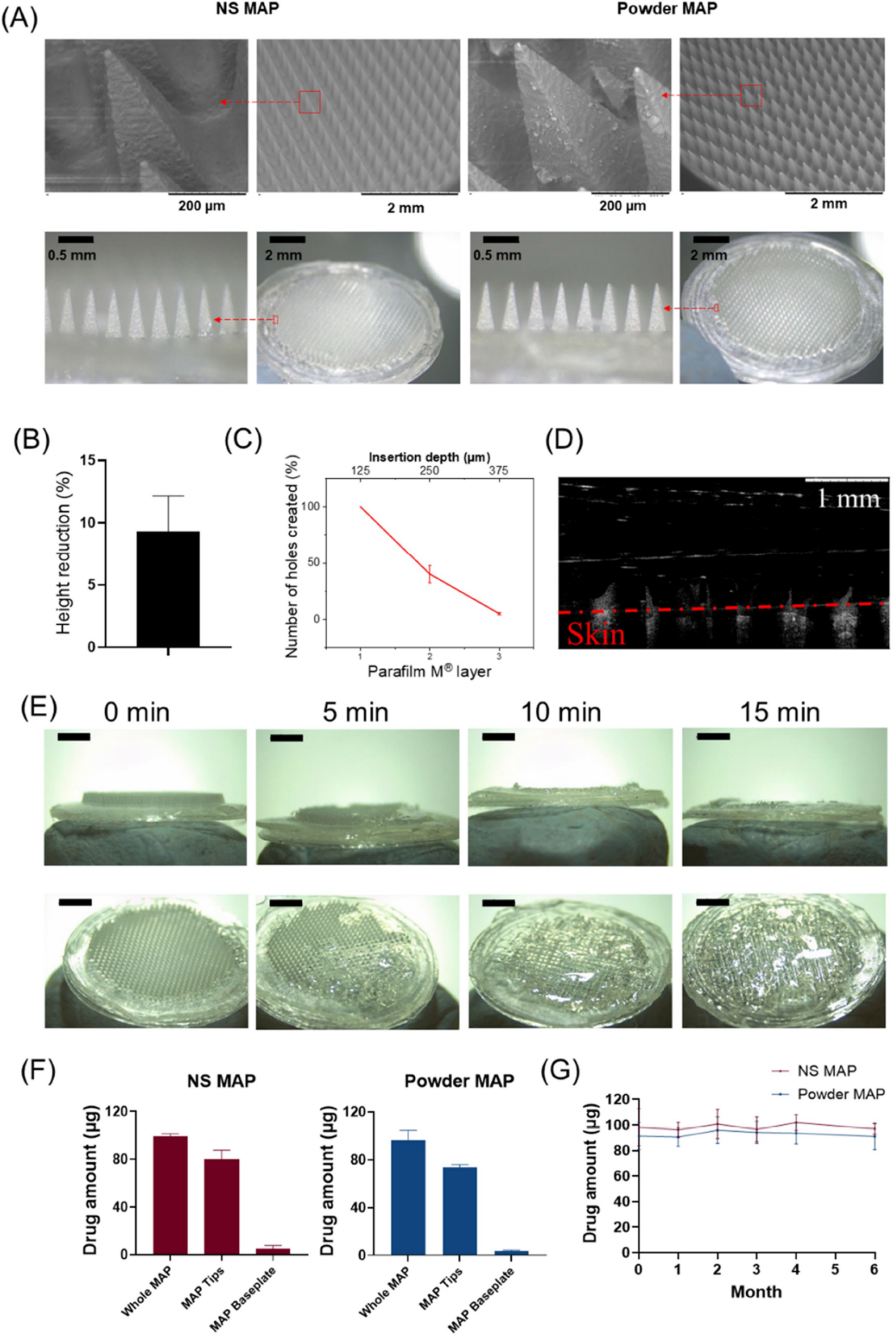
(A) Representative SEM and digital microscopy images of NS MAPs and powder MAPs. (B) Percentage reductions in the height of individual NS microneedles after compression by a force of 32 N/MAP. (C) Insertion of NS MAPs into an artificial skin model consisting of eight layers of Parafilm M with a force of 32 N/MAP. (D) Representative OCT images of NS MAPs following their insertion into full-thickness excised neonatal porcine skin. (E) In-skin dissolution state of NS MAPs. The black scale bar indicates a length of 2 mm, and (F) drug content and distribution of NSs and powder MAPs (mean + SD, *n* = 4). (G) Drug content of CPM MAPs for 6 months (mean ± SD, *n* = 6).

The NS MAP and powder MAP were visualized using a SEM system and a Leica EZ4 light microscope, as shown in Figure 6A. Both MAPs had sharp and smooth tips. The drug was distributed homogeneously, and no obvious aggregation was observed. SEM images revealed that the NS MAPs exhibited improved surface smoothness compared to that of the powder MAPs. This improvement can be attributed to the presence of large micron-sized hydrophobic drug particles in the powder MAPs, whereas the NS MAPs consisted of nanosized particles.

#### 3.3.2 Mechanical Performance and Insertion Behavior of the NS MAP

MAPs must possess sufficient strength to be successfully inserted into the skin without bending or breakage. A compression force of 32 N/array, equivalent to the manual compression force typically applied during MAP insertion into the skin, was employed. In previous studies, the best mechanical and insertion properties were observed when 40% PVA and 40% PVP were mixed in equal amounts. The needle height reduction in the CPM MAPs was 9.29 ± 2.87% (Figure 6B). It has been demonstrated that using eight layers of Parafilm M as a model for human skin is a suitable alternative for studying MAP insertion. Figure 6C shows that the MAPs successfully pierce through the first layer of Parafilm M with a penetration rate of 100 ± 0% (*n* = 3) and reach the depth of the third layer. OCT images revealed the state of MAP inserted into the skin with excised porcine skin. From the representative OCT images (Figure 6D), it can be seen that the resulting MAPs were inserted beneath the skin surface.

#### 3.3.3 Dissolution of the CPM NS MAP

The dissolution time and performance of the lyophilized NS MAPs were evaluated by applying them to *ex vivo* full-thickness neonatal porcine skin and observing them using a light microscope, providing insights into their morphology and dissolution process (Figure 6E). Within 5 min after MAP application, the needles started to dissolve, resulting in a noticeable reduction in needle height. The dissolution process continued over a period of 10 min, and complete dissolution of the tips was achieved in 15 min. Due to the trilayer structure of the MAPs, which had a thin drug-free polymer second layer and an undissolved third layer, the second layer of the CPM MAPs was primarily dissolved, and the CPM MAPs were easily removed from the skin.

#### 3.3.4 Drug Content and Distribution in MAPs

The drug content of the CPM MAPs and the drug distribution between the baseplate and MN tips are shown in Figure 6F. The drug content of the whole CPM NS MAPs was 99.41 ± 1.9 μg/patch, with 80.3 ± 7.3 μg accumulated in the MN tips and 4.80 ± 2.95 μg remaining in the baseplate. For the CPM powder MAPs, the total amount of drug loaded was 96.37 ± 8.22 μg/patch, of which 73.51 ± 2.46 μg was in the MN tips and 3.54 ± 0.80 μg was in the baseplate.

#### 3.3.5 Stability Tests of CPM MAPs

Stability tests of the CPM powder MAPs and CPM NS MAPs were also conducted to evaluate the drug content over a period of 6 months. The results of the test showed that the drug concentration remained stable throughout the entire duration of the study (Figure 6G).

#### 3.3.6 Ex Vivo Skin Deposition and Permeation Studies

**Figure 7:**
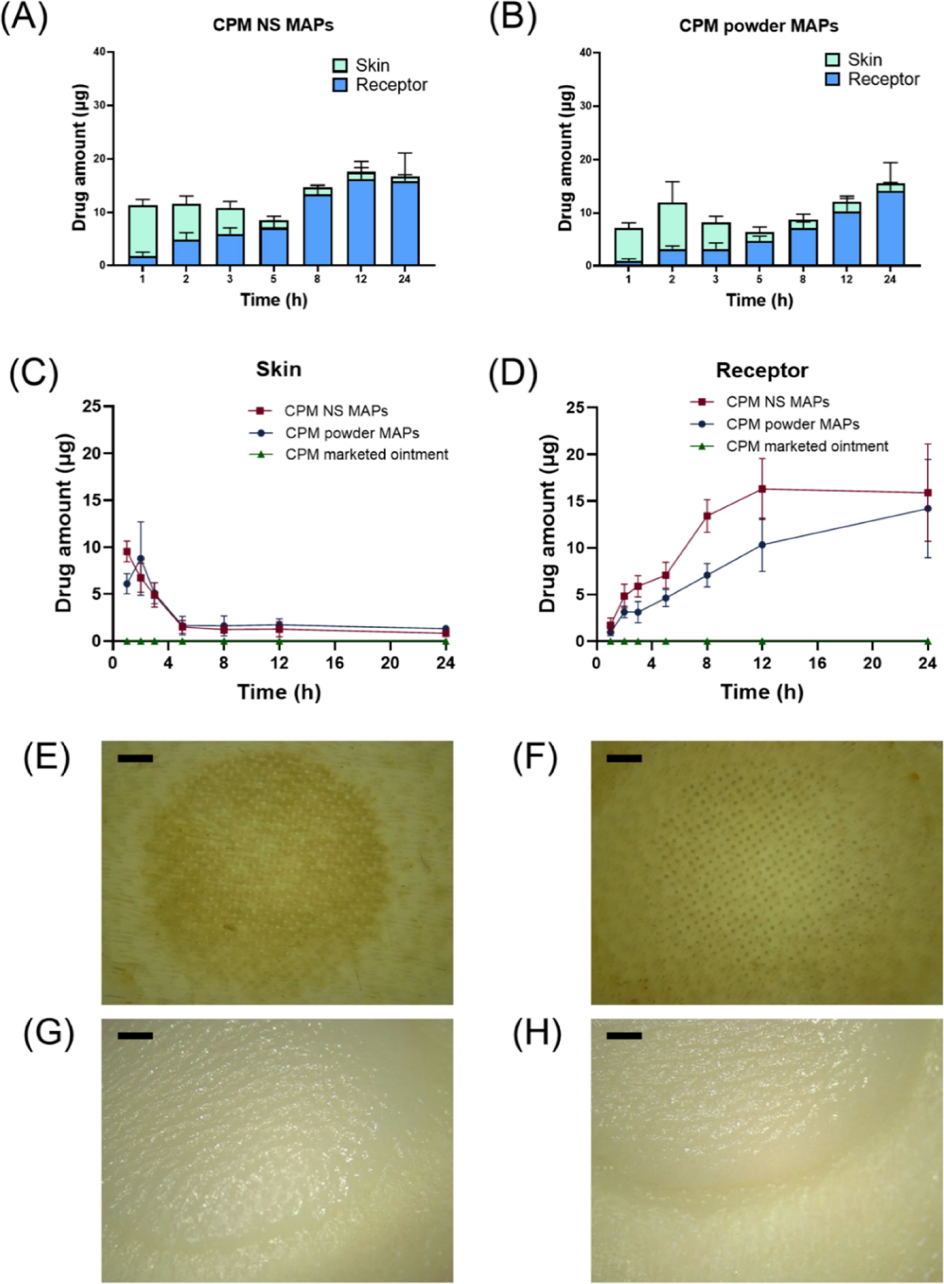
Drug distribution at different times after the application of (A) CPM NS MAPs and (B) CPM powder MAPs (mean + SD, *n* = 4). (C) Drug deposition in the skin after CPM MAP application at different times (mean ± SD, *n* = 4). (D) Drug permeation across full-thickness skin after CPM MAP application at different times (mean ± SD, *n* = 4). Representative digital images of skins after applying (E) CPM NS MAPs, (F) CPM powder MAPs, (G) CPM marketed ointment, or (H) CPM marketed ointment for 24 h. The black scale bar indicates a length of 0.2 μm.

Encapsulated CPM was released from the applied MAPs and dispersed in the skin over time. Ex vivo in-skin deposition and permeation through the skin were evaluated through the Franz cell diffusion setup. Figure 7 illustrates the results of applying CPM MAPs and marketed ointment at various time intervals. The figure shows drug deposition in the skin and drug permeation across the skin in the Franz-cell diffusion model over 24 h. Upon application, more drug was deposited in the skin from the NS MAPs than from the powder MAPs in the first hour, indicating that NSs promote rapid skin deposition. In comparison to powder MAPs, NS MAPs could deliver more drugs to the receptor in 24 h. Both the amounts of CPM in the receptor and the skin from the ointment were lower than the limit of detection (0.15 μg/mL). The skin was observed after the application of MAPs and ointment, as shown in Figure 7E–H. The skin was thicker after the application of the ointment than after the application of the MAPs due to the occlusive property of the ointment matrix.

### 3.4 In Vivo Studies

#### 3.4.1 CPM-Based Measures Reduce Psoriasis Severity

**Figure 8:**
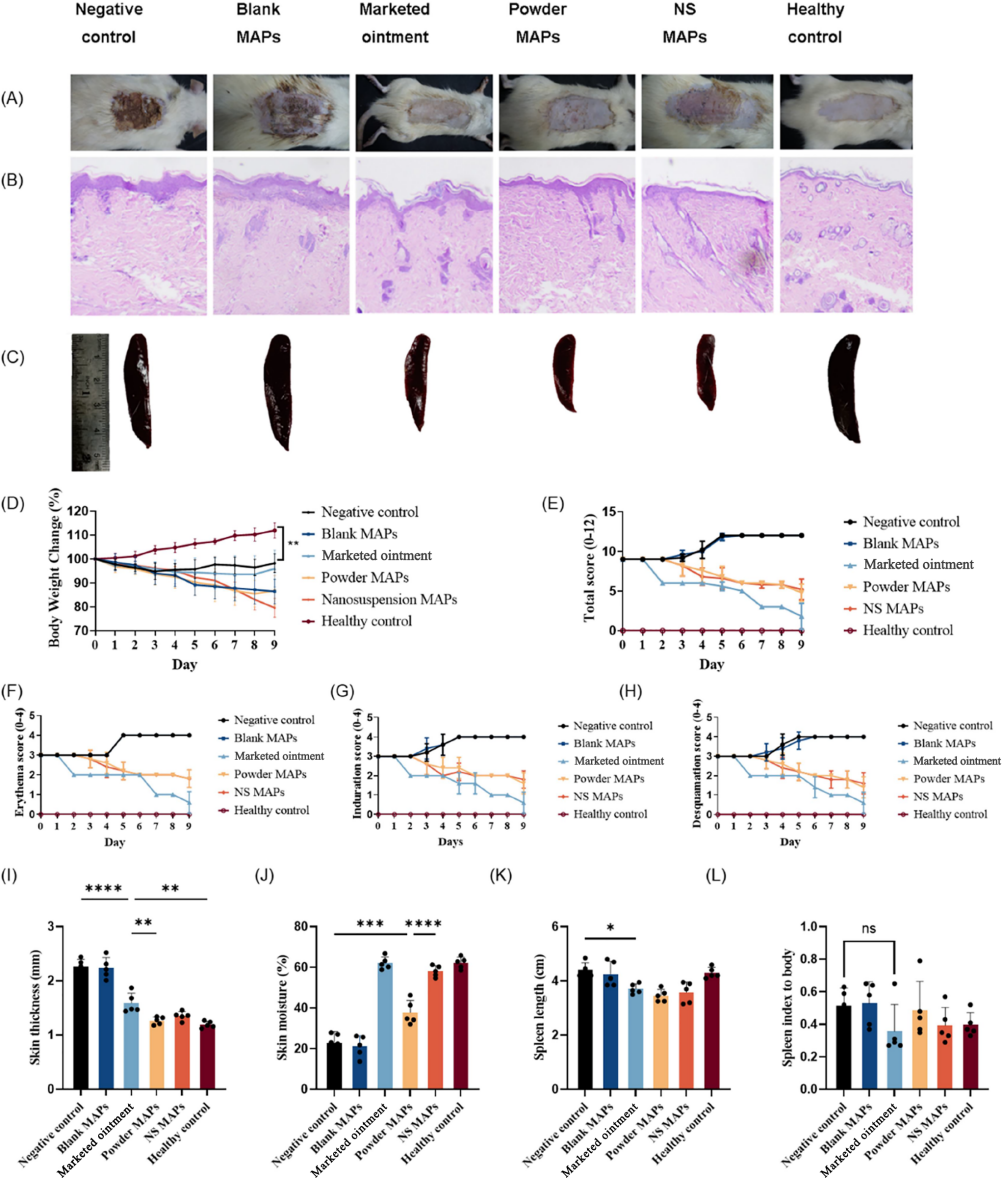
Representative images of (A) skin morphology, (B) H&E staining of the dorsal skin, and (C) spleen from each group of rats. (D) Percentage of body weight change during the therapy period (mean ± SD, *n* = 5). PASI scores were recorded every day until the end of the experiment. (E) Cumulative score (erythema plus induration plus desquamation), (F) erythema score, (G) induration score, and (H) desquamation score (mean ± SD, *n* = 5). (I) Skin thickness, (J) skin moisture at the end of the treatments, (K) spleen length, and (L) spleen index after sacrifice (mean + SD, *n* = 5). Each dot symbol represents an individual rat.

Several scoring systems have been utilized for evaluating plaque-type psoriasis severity. PASI is currently the most extensively validated and commonly used scale for this purpose.^11^ The results for typical skin lesions and PASI scores are shown in Figure 8A,E–H, respectively. The change in body weight of the rats indicated their overall status and physiological metabolism (Figure 8D). Compared with that of healthy rats, the body weight of IMQ-induced psoriatic rats continuously decreased throughout the study. In contrast, after treatment with CPM (for MAPs and ointment), more weight loss was observed. The rats lost weight after the application of MAPs with or without CPM. These findings suggested that with the onset of psoriasis, both CPM and MAPs cause rats to lose weight.

As shown in Figure 8A, rats in the healthy group had a normal back skin appearance, and those in the IMQ-induced group had severe erythema, thickened skin, and desquamation, suggesting that an animal model of psoriasis was successfully established. After CPM treatments (marketed ointment, powder MAPs, and NS MAPs), the severity of psoriasis was noticeably reduced. The slight reduction in psoriasis symptoms with blank MAPs may be because of the moisturization and hydration effects caused by the occlusive dressing. The PASI score is an influential indicator for quantifying psoriasis severity. A higher PASI indicates more extensive lesions and severe skin lesions than a lower PASI score. A psoriasis model was established in rats when all the parameters, namely, erythema, induration, and desquamation, increased to a score of 3 (indicating marked symptoms) (Figure 8E). This point marked Day 0, from which the scoring of symptoms began. After the application of IMQ or without treatment (negative control group and blank MAP group), the severity of clinical signs and psoriasis symptoms in the model rats progressively increased. By day 9, the total symptom score had reached 12. Compared to these untreated groups, the application of CPM MAPs and commercial ointment ameliorated psoriasis severity. After 8 days of treatment, the group treated with CPM powder MAPs had a total score of 4.8. This indicated a 60% decrease from the untreated baseline score of 12. Similarly, the total score on the CPM NS MAP was 5.2, indicating a 57% improvement. The most notable improvement was observed in the group treated with the ointment, which had a total score of 1.8, indicating an 85% reduction from the baseline. All these therapies showed that the treatments were effective.

#### 3.4.2 Changes in Skin Thickness and Moisture

Skin thickening and loss of water are consistent with the severity of psoriasis. The skin thickness and moisture of the rats in each group were measured after sacrifice. As shown in Figure 8I, the mean thickness of the skin in the healthy control group was 1.19 mm, and IMQ treatment resulted in an increase to 2.26 mm (∼1.9-fold thicker) because of acanthosis caused by cellular hyperproliferation. There was no significant difference in skin thickness among the CPM powder MAP group, the CPM NS MAP group, or the healthy control group. The *p* value between the CPM powder MAP group and the CPM NS MAP group was 0.9064, that between the CPM powder MAP group and the healthy control group was 0.9536, and that between the CPM NS MAP group and the healthy control group was 0.4403. These findings suggested that the differences between the CPM powder MAP group and the CPM NS MAP group were not statistically significant, but both were effective at restoring skin thickness to healthy levels, likely through the normalization of cell differentiation and proliferation. The skin thicknesses of the plants in the CPM powder MAP and NS MAP groups were 1.27 and 1.35 mm, respectively, which were greater than those of the commercial ointment treatment group, in which the thickness was 1.59 mm. There was a significant difference between the commercial ointment group and the powder MAP group (*p* = 0.0095), while there was no significant difference between the commercial ointment group and the NS MAP group (*p* = 0.0918). There was a significant difference in skin thickness between the commercial ointment group and the negative control group (*p* < 0.0001) and that between the commercial ointment group and the healthy control group (*p* = 0.0012). Although the ointment significantly reduced the thickness of psoriasis-like skin, it did not restore the skin to the thickness observed in healthy controls.

The percentage of skin moisture was measured, and the results are shown in Figure 8J. Compared with that in the blank MAP treatment group, the decrease in skin moisture induced by IMQ was significantly inhibited by CPM treatment with either MAPs or ointment (*p* < 0.0001). The skin moisture level increased to a healthy level in the CPM NS MAP treatment group (*p* = 0.6972). The skin moisture content of the NS MAPs was significantly greater than that of the powder MAPs (*p* < 0.0001). The higher skin moisture levels observed in the ointment group than in the MAP treatment group could be attributed to the occlusive properties of the ointment. This was substantiated by the increased skin thickness observed above in the *ex vivo* deposition and permeation studies conducted using a Franz cell apparatus setup upon application of the ointment.

#### 3.4.3 Skin Histopathology

To observe the symptoms histologically, H&E staining was performed on the dorsal skin of each group of rats (Figure 8B). Consistent with the findings above, the IMQ and blank microneedle-treated groups showed an increase in epidermal thickness compared to that of the healthy control group, clearly demonstrating well-developed psoriasis after IMQ application. NS MAPs, powder MAPs, and marketed ointment led to the recovery of the skin structure and alleviation of hyperproliferation, with a reduction in epidermal thickness relative to those in the IMQ groups. Histopathology revealed that MAPs have the same effect as ointments in reducing the severity of psoriatic skin symptoms.

#### 3.4.4 Spleen Morphological Changes

The spleen, which accounts for 25% of the total lymphatic tissue in the body, is the largest immune organ and contains a large number of lymphocytes and macrophages, making it the center of cellular and humoral immunity. In addition to skin inflammation, psoriasis can also cause systemic symptoms, including splenomegaly.^43^ Representative images of the spleen are shown in Figure 8C. The negative control group, which only received IMQ administration without CPM therapy, exhibited an enlargement of the spleen, consistent with the findings of previous studies, which attributed this enlargement to the release of inflammatory cytokines.^70^ Treatment with CPM inhibited this effect, and the spleens in the CPM treatment groups, including the ointment and MAP groups, were smaller than those in the negative control group, blank MAP group, and healthy control group. As illustrated in Figure 8K, the negative control group exhibited a mean spleen length of 4.4 cm, while the CPM treatment groups had notably shorter spleens. Among the CPM treatment groups, the powder MAP group had the shortest spleen length (3.26 cm), followed by the NS MAP group (3.58 cm) and the ointment group (3.71 cm). These treatment outcomes were statistically significant, with *p* values of 0.0007, 0.0034, and 0.0169, respectively. The spleen length in the healthy control group was longer than that in the treatment groups. This can be attributed to the weight gain observed in the healthy group rats, in contrast to the weight loss observed in the disease group. The spleen weight index (Figure 8L) increased to 0.52 in the negative control rats compared with that in the healthy rats (0.4), as expected. The blank MAPs did not affect the spleen indices (*p* = 0.9997). All the CPM treatment groups displayed a decrease in the spleen index after treatment, although the difference was not significant (*p* > 0.05). The lowest index was observed in the ointment group, for which the *p* value was 0.2543 compared to that of the negative control group. The indices of the ointment group and NS MAP group were similar to those of the healthy control group. The lack of a significant difference among all groups could be attributed to the small spleen mass relative to the large body mass. These results suggested that the antipsoriatic activity of CPM MAPs was comparable to that of the ointment in terms of spleen recovery.

## 4 Conclusions

The development of calcipotriol NS-loaded dissolving MAPs represents a promising approach for the treatment of psoriasis. Psoriasis is a chronic inflammatory skin disease that requires long-term management, and current topical treatments have limitations, such as poor skin permeability and a low drug delivery rate. The novel trilayer dissolving MAPs containing CPM NSs demonstrated improved drug delivery efficiency compared to commercial ointments. The use of NSs in the form of MAPs allowed for an enhanced drug dissolution rate, increased saturation solubility, and improved deposition on the skin. Characterization studies confirmed the compatibility of CPM with the stabilizer and the preservation of the size of the NSs. The MAPs exhibited fast dissolution tips, favorable mechanical properties, and efficient drug delivery. In vivo studies demonstrated the efficacy of calcipotriol NS-loaded dissolved MAPs in the treatment of psoriasis. In an animal model of psoriasis, dissolving MAPs showed comparable effectiveness to a marketed ointment.

Overall, this study highlights the potential of dissolving MAPs as a novel drug delivery system for the efficient treatment of psoriasis using calcipotriol NSs. This system offers advantages such as improved skin permeability and enhanced patient adherence. Based on these promising proof-of-concept results, further studies are needed to validate the controlled release properties, efficacy and safety of this innovative approach, and its potential for clinical translation in the treatment of psoriasis.

## Supporting Information Available

Methodology for determining the saturation solubility of calcipotriol in various solvents, critical for ensuring sink conditions in drug release studies related to psoriasis treatments using microneedle patches (PDF)
