# Supplementary material for: Calcipotriol Nanosuspension-Loaded Trilayer Dissolving Microneedle Patches for the Treatment of Psoriasis: In Vitro Delivery and In Vivo Antipsoriatic Activity Studies
Source: Mol Pharm. 2024 May 16;21(6):2813–27. doi: 10.1021/acs.molpharmaceut.3c01223 (PMC11151207; doi:10.1021/acs.molpharmaceut.3c01223)
Supplement: Supplementary file 1 — mp3c01223_si_001.pdf [file mp3c01223_si_001.pdf]

Calcipotriol nanosuspension-loaded trilayer dissolving microneedle patches for the treatment of psoriasis: *in vitro* delivery and *in vivo* antipsoriatic activity studies.

Xianbing Dai[a,b], Andi Dian Permana[c], Mingshan Li[a], Habibie[c], Muhammad Nur Amir[c], Ke Peng[a], Chunyang Zhang[a], Haodong Dai[d], Alejandro J Paredes[a], Lalitkumar K. Vora[a*], Ryan F. Donnelly[a*]

[a]School of Pharmacy, Queen's University Belfast, Medical Biology Centre, 97 Lisburn Road, Belfast BT9 7BL, UK.

[b]School of Pharmacy, Jinzhou Medical University, Jinzhou, Liaoning 121001, China.

[c]Faculty of Pharmacy, Universitas Hasanuddin, Makassar 90245, Indonesia

[d]School of Chemistry and Chemical Engineering, Queen's University Belfast, David Keir Building, Stranmillis Road, Belfast BT9 5AG, UK.

Supplementary materials

Saturation solubility determination

To achieve sink conditions for future release studies and determine the drug solubilities of the NSs, we determined the saturation solubility of the drug and lyophilized NSs in the release medium. Due to its hydrophilic properties, Tween 80 and SLS were initially considered cosolvents. The saturation solubility of bulk CPM in PBS solution containing SLS and Tween 80 at concentrations of 0.5% (w/v), 1% (w/v), and 2% (w/v) was determined. However, when testing the concentration of CPM in SLS-PBS solution, two HPLC peaks were observed regardless of the concentration of SDS solution added, indicating that CPM was degraded in this release medium and is not suitable for further studies. When Tween 80 was used as the cosolvent, the solubility of CPM was still too low to meet the sink conditions, as shown in **Table S1** below. Even with 2% (w/v)

Tween 80, the concentration was only 46.57 ± 0.47 µg/mL. Therefore, 2-propanol was selected to promote CPM solubility. Although more 2-propanol could increase the solubility, it should be considered that high concentrations of organic solvent may damage the full-thickness neonatal porcine skin used for future *ex vivo* drug permeation and deposition studies. When PBS:2-propanol was used at 70:30 (v:v) as the release medium, the solubility was 127.62 ± 8.58 µg/mL, which achieved sink conditions. The saturation solubility of lyophilized CPM NS was significantly increased to 233.37 ± 4.47 µg/mL (p < 0.0001), which was 1.83 times higher than that of bulk CPM (**Figure S1**).

Table S1. CPM saturation solubility in PBS solution containing different concentrations of Tween 80. (Means ± SDs, n=3)

The concentration of Tween 80 (w/v)	0.5%	1%	2%
CPM concentration (µg/mL)	11.79 ± 0.05	23.85 ± 0.16	46.57 ± 0.47

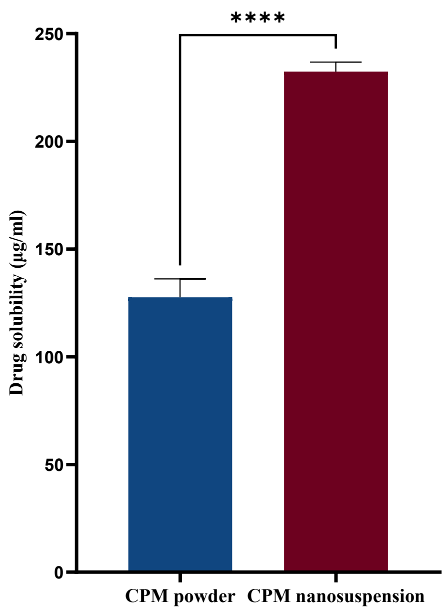

Figure S1. Saturation solubility of CPM in desired release medium. (Means ± SDs, n=3).
